# YM155 as an inhibitor of cancer stemness simultaneously inhibits autophosphorylation of epidermal growth factor receptor and G9a-mediated stemness in lung cancer cells

**DOI:** 10.1371/journal.pone.0182149

**Published:** 2017-08-07

**Authors:** Chun-Chia Cheng, Jungshan Chang, Stanley Ching-Cheng Huang, Huan-Chau Lin, Ai-Sheng Ho, Ken-Hong Lim, Chun-Chao Chang, Ling Huang, Yu-Cheng Chang, Yi-Fang Chang, Cheng-Wen Wu

**Affiliations:** 1 Division of Hematology and Oncology, Department of Internal Medicine, MacKay Memorial Hospital, Taipei, Taiwan; 2 Laboratory of Good Clinical Research Center, Department of Medical Research, MacKay Memorial Hospital, Tamsui District, New Taipei City, Taiwan; 3 Institute of Clinical Medicine, National Yang-Ming University, Taipei, Taiwan; 4 Graduate Institute of Medical Sciences, School of Medicine, College of Medicine, Taipei Medical University, Taipei, Taiwan; 5 Department of Pathology & Immunology, Washington University School of Medicine, St. Louis, Missouri, United States of America; 6 Division of Gastroenterology, Cheng Hsin General Hospital, Taipei, Taiwan; 7 Division of Gastroenterology and Hepatology, Department of Internal Medicine, Taipei Medical University Hospital, Taipei, Taiwan; 8 Division of Gastroenterology and Hepatology, Department of Internal Medicine, School of Medicine, College of Medicine, Taipei Medical University, Taipei, Taiwan; Seoul National University College of Pharmacy, REPUBLIC OF KOREA

## Abstract

Cancer stem cell survival is the leading factor for tumor recurrence after tumor-suppressive treatments. Therefore, specific and efficient inhibitors of cancer stemness must be discovered for reducing tumor recurrence. YM155 has been indicated to significantly reduce stemness-derived tumorsphere formation. However, the pharmaceutical mechanism of YM155 against cancer stemness is unclear. This study investigated the potential mechanism of YM155 against cancer stemness in lung cancer. Tumorspheres derived from epidermal growth factor receptor (EGFR)-mutant HCC827 and EGFR wild-type A549 cells expressing higher cancer stemness markers (*CD133*, *Oct4*, and *Nanog*) were used as cancer stemness models. We observed that EGFR autophosphorylation (Y1068) was higher in HCC827- and A549-derived tumorspheres than in parental cells; this autophosphorylation induced tumorsphere formation by activating G9a-mediated stemness. Notably, YM155 inhibited tumorsphere formation by blocking the autophosphorylation of EGFR and the EGFR-G9a-mediated stemness pathway. The chemical and genetic inhibition of EGFR and G9a revealed the significant role of the EGFR-G9a pathway in maintaining the cancer stemness property. In conclusion, this study not only revealed that EGFR could trigger tumorsphere formation by elevating G9a-mediated stemness but also demonstrated that YM155 could inhibit this formation by simultaneously blocking EGFR autophosphorylation and G9a activity, thus acting as a potent agent against lung cancer stemness.

## Introduction

The epidermal growth factor receptor (EGFR) overexpresses and activates the downstream phosphoinositide 3-kinase-AKT and mitogen-activated protein kinase-extracellular signal-regulated kinase (ERK; MEK) signaling pathways, respectively, thus regulating the survival and proliferation of cancer cells [1,2]. EGFR is particularly overexpressed in lung cancer. EGFR mutations have been reported to constitutively cause EGFR autophosphorylation [3,4]; the resulting poor patient survival rate is the major reason for drug resistance and tumor recurrence. EGFR overexpression exceeds 90% in lung cancer, acting as a target for potent therapeutic agents. Therefore, we hypothesized that EGFR participates in maintaining the cancer stemness property as the leading cause of tumor recurrence.

To test the hypothesis, we used YM155 to reduce tumorsphere formation derived from EGFR-positive lung cancer cells, because YM155 was demonstrated to suppress EGFR in pancreatic cells by degrading it [5]. We also investigated the detailed molecular mechanisms of YM155 as a potent inhibitor of cancer stemness cells (CSCs). YM155 was reported to be capable of reducing cancer stemness in gastric cancer [6]. In addition, the structure of YM155 is similar to that of a stemness inhibitor, BBI608 [7,8]. This evidence reveals that YM155 is a potent agent against lung cancer stemness. YM155 is an imidazolium-based survivin-suppressing compound that binds to interleukin enhancer-binding factor 3 (ILF3) [9] and inhibits a member of the inhibitor apoptosis protein [10]. Although YM155 inhibits anti-apoptosis by suppressing ILF3-mediated survivin, its detailed mechanism in the inhibition of cancer stemness is unclear.

In clinical practice, afatinib, a tyrosine kinase inhibitor (TKI), has been reported to inhibit EGFR phosphorylation and further suppress tumor progression [11–13]. Wang et al revealed that afatinib enhances the therapeutic efficacy of chemotherapeutic agents by suppressing CSCs [14]. Therefore, we proposed the hypothesis that the EGFR-mediated downstream pathway in the initiation of CSCs may be a target for discovering potent tumor therapeutic agents against cancer stemness. CSCs have been demonstrated to survive many tumor repressive treatments [15,16]. Post-translational modifications are critical for regulating the cellular function and phenotypic expression of cancer cells [17], thus leading to an immediate rescue of the cells under stressful therapeutic conditions. Furthermore, EGFR promotes chromatin condensation through methylation on H3K9 in KRAS-mutant lung A549 cells in response to stressful ionizing radiation [18]. Therefore, we proposed that the stemness property could be regulated by EGFR-mediated epigenetic modifications for maintaining the undifferentiating tumor status. Epigenetic modification enzymes, histone methyltransferases (HMTs) containing the SET domain, have been demonstrated to facilitate gene expression or silencing [19,20], thus possessing bivalent functions through methylation on histone 3 lysine residues. Among the HMTs, G9a, involved in embryonic development [21], was considered to maintain the stemness property in this study. G9a mainly methylates on histone 3 lysine 9 (H3K9) to regulate the transcriptional genes through gene silencing [22]. G9a can maintain CSC characteristics in head and neck squamous cell carcinoma [23]; therefore, we investigated whether G9a is generated as the downstream protein of EGFR in EGFR-positive lung tumor cells, for determining the stemness property in EGFR-positive lung cancer.

In this study, we established HCC827- and A549-derived CSC formation models and investigated the possible pharmaceutical mechanisms of YM155 against lung cancer stemness. We found that increased EGFR phosphorylation resulted in G9a upregulation in HCC827- and A549-derived tumorspheres to maintain cancer stemness. YM155 efficiently inhibited EGFR autophosphorylation and the EGFR-mediated G9a stemness pathway simultaneously, thus rendering it a potential agent against lung cancer stemness.

## Materials and methods

### Cell culture and tumorsphere formation

The lung cancer cell lines HCC827 and A549 were purchased from the American Type Culture Collection (ATCC, Manassas, VA, USA). The cell line H520 was purchased from Bioresource Collection and Research Center (BCRC, Hsinchu, Taiwan) and was authenticated through short tandem repeat profiling by BCRC; it was free of *Mycoplasma*. HCC827 and A549 were used for tumorsphere formation and Western blotting, and they were reauthenticated through short tandem repeat profiling (Applied Biosystems, Massachusetts, USA). The HCC827 and H520 cell lines were cultured in RPMI-1640 medium with 10% fetal bovine serum (FBS) and 1% penicillin–streptomycin. A549 was cultured in Dulbecco’s modified Eagle medium (DMEM) with the same additives. For tumorsphere formation, the cells were cultured in low-attached 6-well plates with serum-free medium containing B27 (Invitrogen, Massachusetts, USA), 20 ng/mL of EGF (Sigma, Missouri, USA), 20 ng/mL of fibroblast growth factor (bFGF, Sigma), 5 μg/mL of bovine insulin (Sigma), and 4 μg/mL of heparin (Sigma) [24]. All cells were incubated at 37°C and 5% of CO_2_. Cancer-initiating and early progenitor cells survived and proliferated, but differentiated cells died [25]. The cells were observed using an inverted microscope.

### mRNA extraction and cDNA preparation

The HCC827 cells and HCC827 CSCs were cultured in a 6-cm dish for mRNA extraction and harvested using 1 mL of TRIzol (Thermo Fisher Scientific, Massachusetts, USA). The solution was mixed with 200 μL of 1-bromo-3-chloropropane (Sigma), vortexed, and incubated for 5 min at room temperature. The supernatant was collected after 13,000-rpm centrifugation for 15 min at 4°C. Isopropanol (500 μL) was added and incubated for 5 min at room temperature. The pellet was collected after 13,000-rpm centrifugation for 10 min at 4°C. Subsequently, the pellet was incubated with 1 mL of 70% ethanol and centrifuged at 7500 rpm for 10 min at 4°C. Furthermore, the mRNA pellet was dissolved in double-distilled water after air drying. To obtain cDNA, 1 μg of mRNA, 2 μL of random hexamers, and 10 μL of double-distilled water were mixed in a polymerase chain reaction (PCR) tube and incubated at 65°C for 10 min, followed by cooling at 4°C. The solution was mixed with 4 μL of buffer (5×), 0.5 μL of RNase, 2 μL of dNTP (2.5 mM), and 0.5 μL of reverse transcriptase, and it was consequently treated at 25°C for 10 min, 50°C for 1 h, and 85°C for 5 min, followed by cooling at 4°C.

### Quantitative PCR

Quantitative PCR (Applied Biosystems, California, USA) was performed using the SYBR Green system (Applied Biosystems, California, USA) according to the manufacturer’s instruction. The following primers were used. *Aldehyde dehydrogenase 1 (Aldh1)*: 5′-GTGGATTCAAGATGTCTGGAAATG-3′ and 5′-ATATTAGTGACTGTAAGGAGATGCT-3′. *CD133*: 5′-CTATTCAGGATATACTCTCAGCATT-3′ and 5′-TTTCTGTGGATGTAACTTTCAGTG-3′. *Oct4*: 5′-AAAGCAGAAACCCTCGT-3′ and 5′-TCCAGGTTGCCTCTCAC-3′. *Nanog*: 5′-GAGACAGAAATACCTCAGCC-3′ and 5′-TCTGCGTCACACCATTG-3′. *G9a*: 5′-GGGAGGAGCTAGGGTTTGAC-3′ and 5′-TGTGGTCCGTTCTCATGTGT-3′. *GLP*: 5′-CCAGTGCATGGCTACAGAGA-3′ and 5′-AGAAGTAGCCACAGCCAGGA-3′. *SUV39H1*: 5′-GGCAACATCTCCCACTTTGT-3′ and 5′-CAATACGGACCCGCTTCTTA-3′. *SUV39H2*: 5′-GATTTAGAGGGCCCACCTTC-3′ and 5′-GGGAGTACCAGGTGGGATTT-3′. *SETDB1*: 5′-TCCCAGGATCTGCATAAAGG-3′ and 5′-TCAGCAGGAGGGTGGTAATC-3′. *SETDB2*: 5′-AGATGTAACCAGGCGACCAC-3′ and 5′-TTCCTTCTTTTGTGGCATCC-3′. *GAPDH*: 5′-GAGTCAACGGATTTGGTCGT-3′ and 5′-TTGATTTTGGAGGGATCTCG-3′.

### Gene knockdown

*EGFR* and *G9a* knockdown was conducted using a short-hairpin RNA (shRNA)-expression lentivirus system that contains the specific shRNA in the vector pLKO.1-puro generated in 293T cells. The *EGFR* and *G9a* pLKO plasmid and scrambled control were purchased from National RNAi Core Facility of Academia Sinica, Taipei, Taiwan. For producing lentivirus, 293T cells (70% confluence) cultured in DMEM containing 10% FBS and 0.1% penicillin–streptomycin (6-cm dish) were transfected with 4 μg of *EGFR* or *G9a* pLKO.1 vectors, 1 μg of the envelope plasmid pVSV-G, and 3.6 μg of the packaging plasmid pCMVΔR8.91. The plasmids were preincubated with 400 μL of Lipofectamine 2000 for 20 min at room temperature and consequently added to 293T cells. The cultured medium was substituted with fresh DMEM containing 30% FBS and 1% of penicillin–streptomycin and incubated for 4 h. The virus solution was collected after 48 h of transfection and stored at −80°C. HCC827 or A549 cells cultured in 80% confluence were infected with the prepared lentivirus (preincubated with 8 μg/mL of polybrene) for 24 h. The cells were then changed with RPMI-1640 medium for HCC827 cells or DMEM for A549 cells containing 10% FBS, 1% penicillin–streptomycin, and 2 μg/mL of puromycin, which were harvested after 48 h.

### Western blotting

The cells were lysed in RIPA buffer containing 50 mM Tris-HCl (pH 7.4), 1% NP-40, 0.5% Na-deoxycholate, 0.1% sodium dodecyl sulfate (SDS), 2 mM ethylenediaminetetraacetic acid, 50 mM NaF, and 150 mM NaCl. The lysed proteins were mixed with 5× sample buffer [75 mM Tris-HCl, pH 6.8, 10% (v/v) glycerol, 2% SDS (w/v), 0.002% (w/v) bromophenol blue]. In total, 20 μg of each sample was analyzed through 10% SDS-polyacrylamide gel electrophoresis and then transferred onto Immobilon-P polyvinylidene fluoride (PVDF) membranes (Merck Millipore, Massachusetts, USA). These membranes were blocked with 5% skim milk for 1 h at room temperature, incubated with primary antibodies (1 μg/mL) overnight at 4°C, and washed using Tris-buffered saline with 0.1% Tween-20. The specific antibodies against EGFR, pEGFR (Y1068), and mH3K9 were purchased from Cell Signaling (Danvers, Massachusetts, USA), and Oct4 and G9a were purchased from Novus Biologicals (Littleton, Colorado, USA). After washing, the PVDF membranes were incubated with horseradish peroxidase-conjugated secondary antibody (1 μg/mL) for 2 h at room temperature. The immunoreactive proteins were detected through an enhanced chemiluminescence kit (Bio-Rad, California, USA) coupled with an LAS-4000 mini device (Fujifilm, Tokyo, Japan).

### Cell viability

The WST-1 (2-(4-iodophenyl)-3-(4-nitrophenyl)-5-(2,4-disulfophenyl)-2H-tetrazolium, monosodium salt; Takara) assay was used to determine cell viability after incubation with YM155, afatinib, and UNC0642 for 48 h. At least three replicates were performed.

### Statistical analysis

Statistical analyses were performed using GraphPad Prism V5.01 (GraphPad Software, Inc., California, USA). All analytical data with more than two groups were evaluated using analysis of variance, followed by post hoc analysis with Bonferroni’s test. Student’s t test was used to compare two groups. Moreover, *p* < 0.05 was considered to indicate a statistically significant difference.

## Results

### Elevated autophosphorylation (Y1068) of EGFR and methylation on H3K9 in HCC827-formed tumorspheres

**Fig 1:**
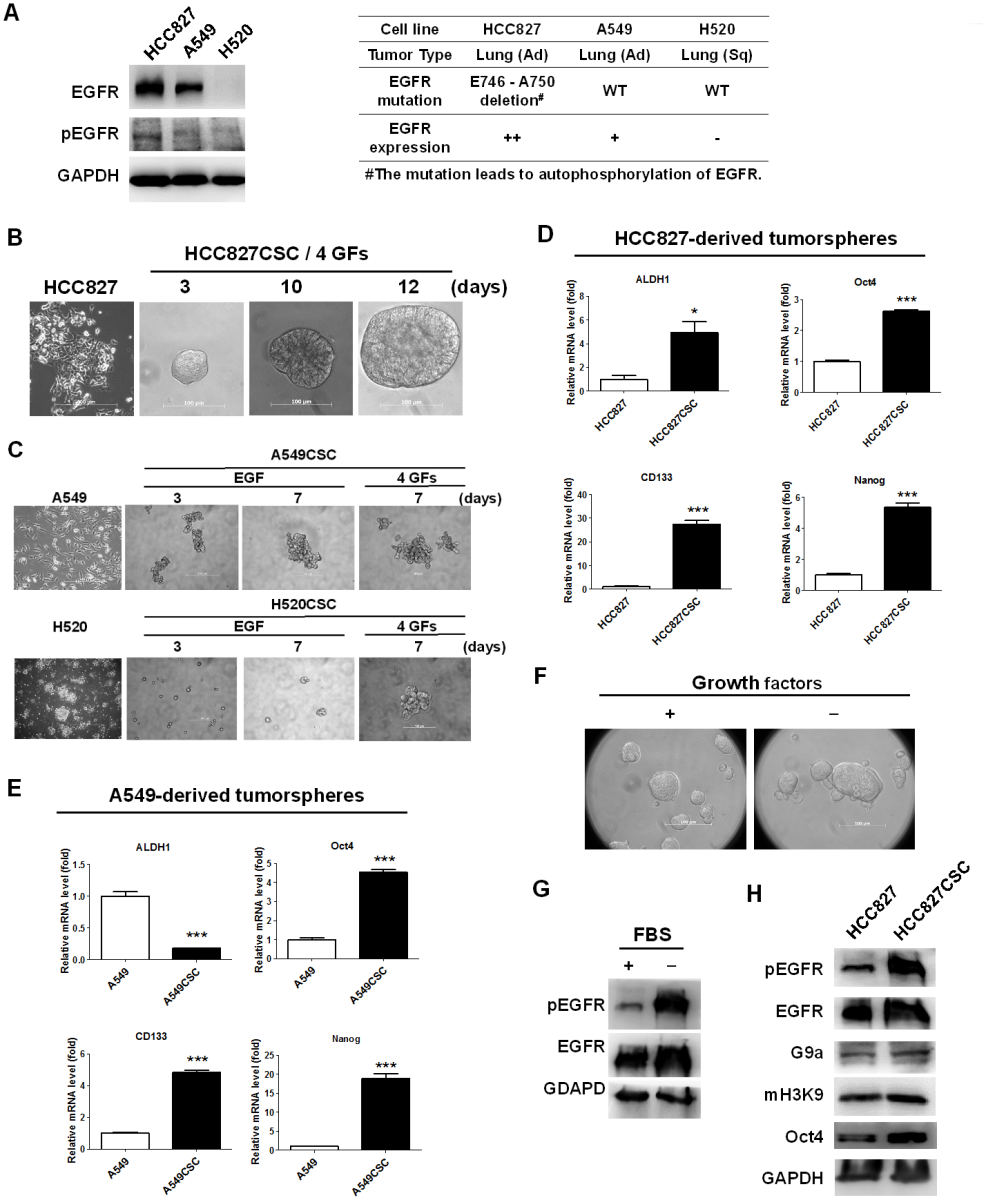
Elevated phosphorylation of EGFR and G9a-mediated methylation on H3K9 in HCC827-derived CSCs. (A) HCC827^++^, A549^+^, and H520^−^ were selected for analyzing EGFR phosphorylation. (++) high expression; (+) moderate expression; (−) no expression. The results indicated higher EGFR expression in HCC827 cells than in A549 and H520 cells, whereas autophosphorylation of EGFR in HCC827 cells. (B and C) HCC827- or A549-derived tumorspheres were detected and imaged through inverted microscopy when the cells were cultured in a low attached dish with FBS-free B27 supplement medium. For triggering the formation of temporary CSCs, four additive growth factors (4 GFs) were added, namely EGF, bFGF, insulin, and heparin. (D) HCC827- and A549-derived tumorspheres were investigated to detect the expression of stemness markers, *ALDH1*, *CD133*, *Oct4*, and *Nanog*, for identifying the stemness property. These markers were increased in HCC827 CSCs (n = 3) compared with parental HCC827 cells (n = 3), but (E) only *CD133*, *Oct4*, and *Nanog* were increased in A549 CSCs. (F) In addition, HCC827 cells cultured in serum-free B27 supplement medium without the aforementioned factors formed tumorspheres with (G) elevated EGFR phosphorylation. (H) EGFR phosphorylation was higher in HCC827 CSCs than in parental HCC827 cells, accompanied by higher G9a expression and H3K9 methylation, whereas *Oct4* was used as a stemness marker. Scale bar: 100 μm. *p < 0.05 and ***p < 0.001.

To investigate the molecular mechanism of CSCs in EGFR-positive lung cancer, we first examined the expression of EGFR in three lung cancer cell lines, namely HCC827, A549, and H520. HCC827 (EGFR E746-A750 deletion) and A549 (EGFR wild-type) are adenocarcinomas, whereas H520, an EGFR-negative cell line, is a squamous carcinoma of the lung (Fig 1A). Western blotting revealed higher EGFR expression and autophosphorylation in HCC827 cells than in A549 and H520 cells (Fig 1A). HCC827 cells were adherent but could form a tumorsphere exceeding 100 μm in 12 days (Fig 1B) when cultured in a low-attached dish with serum-free medium, as described in the Materials and Methods section. A549 (EGFR-positive) and H520 (EGFR-negative) cells could also form tumorspheres in 7 days in the addition of 4 growth factors, EGF, FGF, insulin, and heparin (Fig 1C), whereas, EGF only triggered A549 to form tumorspheres (Fig 1C). We further identified the stemness characteristics in the formed tumorspheres through quantitative reverse transcription PCR by measuring the expression of cancer stemness markers, namely *Aldh1*, *Cd133*, *Oct4*, and *Nanog*. The results indicated higher expression levels of *Aldh1*, *Cd133*, *Oct4*, and *Nanog* in the tumorspheres (HCC827 CSCs) than in parental HCC827 cells (Fig 1D). Moreover, A549 CSCs expressed higher mRNA levels of *Cd133*, *Oct4*, and *Nanog* but exhibited reduced mRNA levels of *Aldh1* (Fig 1E). Both stemness models were used to investigate the cellular stemness property of EGFR-positive lung cancer. We subsequently investigated the growth factors (EGF, FGF, insulin, and heparin in the FBS-free B27-supplement medium) that majorly regulate the formation of HCC827 tumorspheres. We cultured HCC827 cells in the medium without EGF, FGF, insulin, or heparin and investigated tumorsphere formation. Notably, HCC827 formed tumorspheres even in the absence of growth factors in the culture (Fig 1F). In addition, we cultured HCC827 cells in the FBS-free B27-supplement medium without growth factors and determined the phosphorylation of EGFR on Y1068 through Western blotting. According to our review of the relevant literature, the autophosphorylation of EGFR on Y1068 can lead to the activation of the granzyme B 2-RAS-RAF-MEK1-ERKs and Janus activated kinase-signal transducer and activator of transcription 3 pathways [26]. Moreover, pY1068 was suggested to be a predictive biomarker for EGFR-TKI treatment [27]. Therefore, this phosphorylated site on Y1068 of EGFR was determined for representing the activation of EGFR. The phosphorylation of EGFR was increased in HCC827 cells cultured in the serum-free medium without growth factors (Fig 1G), suggesting that EGFR activation was the temporary cellular response to the environmental stress under a serum-free condition, which may result in tumorsphere formation. In addition, the HCC827-derived tumorspheres presented higher levels of EGFR phosphorylation, methylation on H3K9, and Oct4 expression than did parental HCC827 cells (Fig 1D), whereas Oct4 was used as a stemness marker.

### YM155 significantly blocked the formation of tumorspheres and inhibited phosphorylation of EGFR and expression and activity of G9a

**Fig 2:**
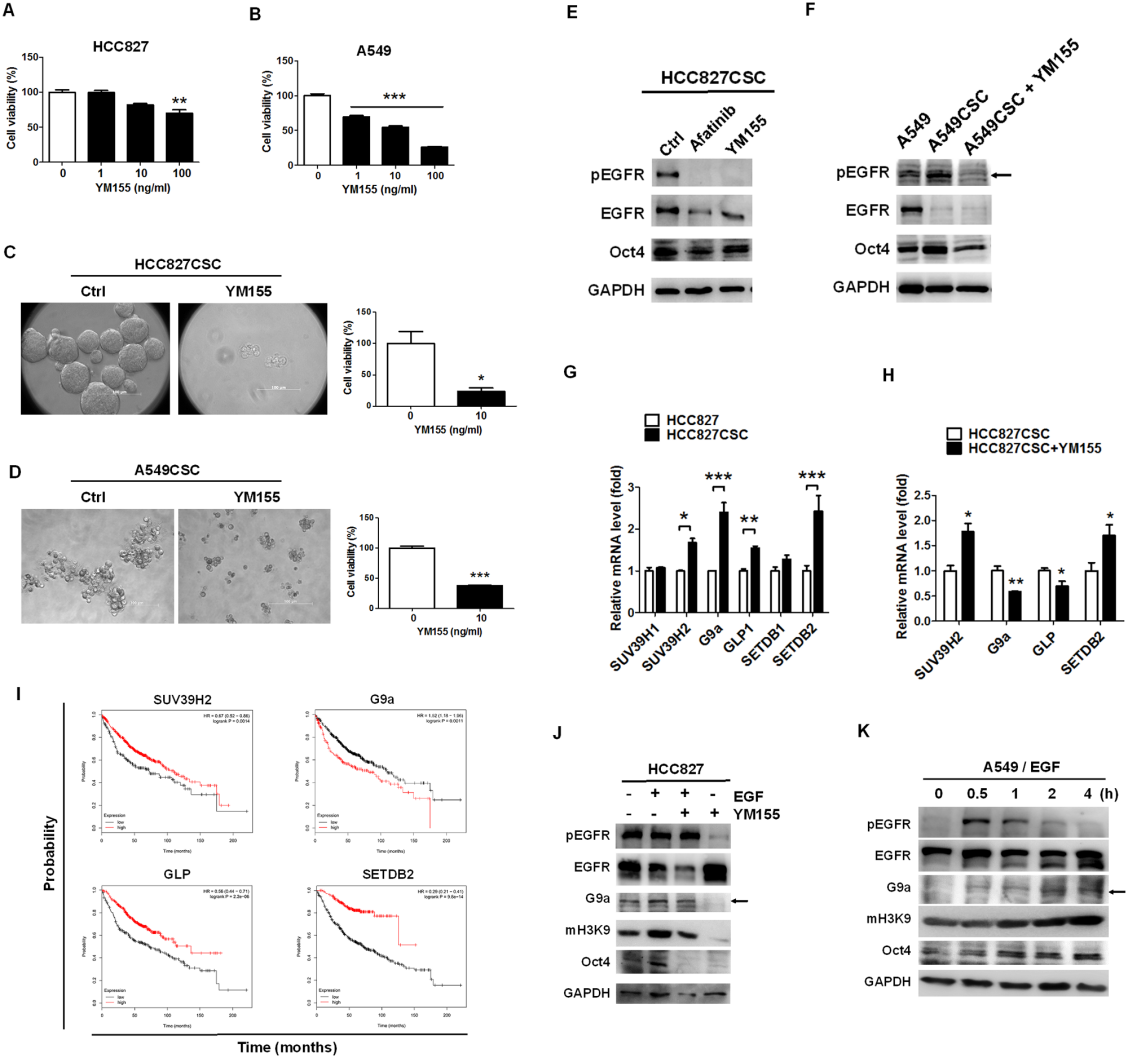
YM155 significantly reduced tumorsphere formation and inhibited EGFR autophosphorylation and G9a expression. YM155 is reported to suppress cancer stemness; therefore, we used this compound to investigate the cellular mechanism of tumorsphere formation. To compare the cytotoxic capacity of YM155 against tumorsphere formation and parental cell lines, YM155 was applied to HCC827 and A549 cells in the stemness cultured or integrated medium. (A) YM155 significantly reduced HCC827 cells when used at a dose of 100 ng/mL (**p < 0.01) and (B) A549 cells when used at a dose of 1 ng/mL (***p < 0.001). (C) Microscopy revealed that 10 ng/mL of YM155 considerably inhibited the formation of HCC827 and (D) A549CSC tumorspheres and resulted in a lower cell viability. (E) Because EGFR overexpresses in HCC827 and A549 cells, and because YM155 has been reported to suppress EGFR, we investigated the autophosphorylation status of EGFR in HCC827- and A549-derived tumorspheres. EGFR phosphorylation was blocked by YM155 in HCC827-derived tumorspheres, whereas afatinib, a tyrosine kinase inhibitor, was used as a control. (F) Moreover, YM155 inhibited EGFR autophosphorylation in A549-derived tumorspheres and reduced *Oct4* expression. (G) The methylation on H3K9 was high in HCC827-derived tumorspheres; therefore, we investigated the mRNA levels of the SET domain-containing proteins, particularly those functioning in H3K9 methylation, in HCC827 CSCs compared with parental HCC827 cells. The results revealed that the mRNA of *SUV39H2*, *G9a*, *GLP*, and *SETDB2* increased in HCC827 CSCs. (H) In addition, YM155 significantly reduced the mRNA levels of *G9a* and *GLP* but increased those of *SUV39H2* and *SETDB2*. (I) To confirm the significant roles of tumorsphere-expressed *SUV39H2*, *G9a*, *GLP*, and *SETDB2* in lung adenocarcinoma, the Kaplan–Meier method was used for investigating the relationship between the mRNA levels of *SUV39H2*, *G9a*, *GLP*, and *SETDB2* and the survival rate in 2,437 patients with lung cancer; the mean follow-up period was 49 months. The results revealed that an elevated mRNA level of *G9a* was associated with a lower overall survival rate in the patients with lung adenocarcinoma, but the remaining three genes yielded controversial results. Higher gene expression levels are indicated in red. (J) We investigated whether EGFR could downregulate *G9a* expression in HCC827 cells. We found that YM155 reduced EGF-induced *G9a* expression, H3K9 methylation, and *Oct4* expression in HCC827 cells. HCC827 cells were treated with 20 ng/mL of EGF with or without 10 ng/mL of YM155. (K) HCC827, an EGFR-mutant strain, can induce autophosphorylation; therefore, we investigated and validated EGFR-regulated *G9a* expression in EGFR wild-type A549 cells. A549 cells treated with 20 ng/mL of EGF expressed immediate EGFR autophosphorylation in 0.5 h and subsequent *G9a*, mH3K9, and *Oct4* expression. The results indicated that EGF induced the expression of *G9a* and *Oct4* in lung cancer. Scale bar: 100 μm. *p < 0.05.

To elucidate and investigate the possible mechanism of the stemness property in EGFR-positive lung cancer, we used YM155, a stemness inhibitor, and investigated its cellular mechanism and potential as an inhibitor of cancer stemness in EGFR-derived cancer stemness models. YM155 was treated with HCC827 and A549 cells in a dose-dependent manner for investigating its cytotoxic capacity in parental lung cancer cells. The results revealed that more than 100 ng/mL of YM155 could reduce the viability of HCC827 cells (Fig 2A). Moreover, 1 ng/mL of YM155 was more sensitive to A549 cells and led to similar inhibition in cell viability compared with HCC827 cells (Fig 2B). Furthermore, 10 ng/mL of YM155 markedly reduced the size of HCC827-derived tumorspheres and resulted in lower cell viability (Fig 2C), in addition to inhibiting A549-derived tumorsphere formation (Fig 2D). Considering the inhibitory activity of YM155 in parental HCC827 and A549 cells, YM155 seemed to perfer to inhibit the growth of tumorspheres owing to its higher capacity to inhibit the viability of tumorspheres at 10 ng/mL.

We found that YM155 inhibited the autophosphorylation of EGFR in HCC827 (Fig 2E) and A549 (Fig 2F) CSCs, resulting in the reduction in *Oct4* expression. Afatinib was used as a positive control to inhibit the phosphorylation of EGFR, which also resulted in the reduction in *Oct4* expression in HCC827 CSCs (Fig 2E). Next, to explore the EGFR-downstream regulators involved in the cancer stemness property, we investigated whether SET domain-containing proteins, which can methylate on H3K9, were associated with maintaining this property. We measured the expression of the SET domain-containing proteins *SUV39H1*, *SUV39H2*, *G9a*, *GLP*, *SETDB1*, and *SETDB2*. The results revealed that the expression levels of *SUV39H2*, *G9a*, *GLP*, and *SETDB2* were significantly increased in HCC827 CSCs compared with parental HCC827 cells (Fig 2G). In addition, YM155 treatment reduced *G9a* and *GLP* expression (Fig 2H), implying that YM155 regulates *G9a* and *GLP* expression in HCC827 CSCs.

In clinical practice, *G9a* mRNA levels were associated with a poor survival rate in lung adenocarcinoma (*p* = 0.0011), as analyzed using the MatInspector program based on The Cancer Genome Atlas (TCGA) database (Fig 2I) [28]. This result indicates that *G9a* is oncogene among *SUV39H2*, *GLP*, *and SETDB2*. We then investigated whether EGFR could regulate *G9a* expression and explored the regulatory effects of YM155 in EGFR-positive HCC827 cells co-treated with EGF. We observed that YM155 reduced the autophosphorylation of EGFR and the expression and activity of G9a in HCC827 cells (Fig 2J), accompanied by reduced *Oct4* expression (Fig 2J). However, YM155 did not inhibit the autophosphorylation of EGFR in EGF co-treatment cells (Fig 2J). A549 cells were subsequently used for investigating the EGFR-G9a regulatory pathway. We treated A549 cells with 20 ng/mL of EGF in a time-dependent manner. The results revealed that EGF induced EGFR phosphorylation in 0.5 h, consequently resulting in the expression of G9a and Oct4, whereas H3K9 methylation served as an activation marker of G9a (Fig 2K).

### Inhibition of EGFR reduced expression and activity of G9a and HCC827-derived tumorsphere formation

**Fig 3:**
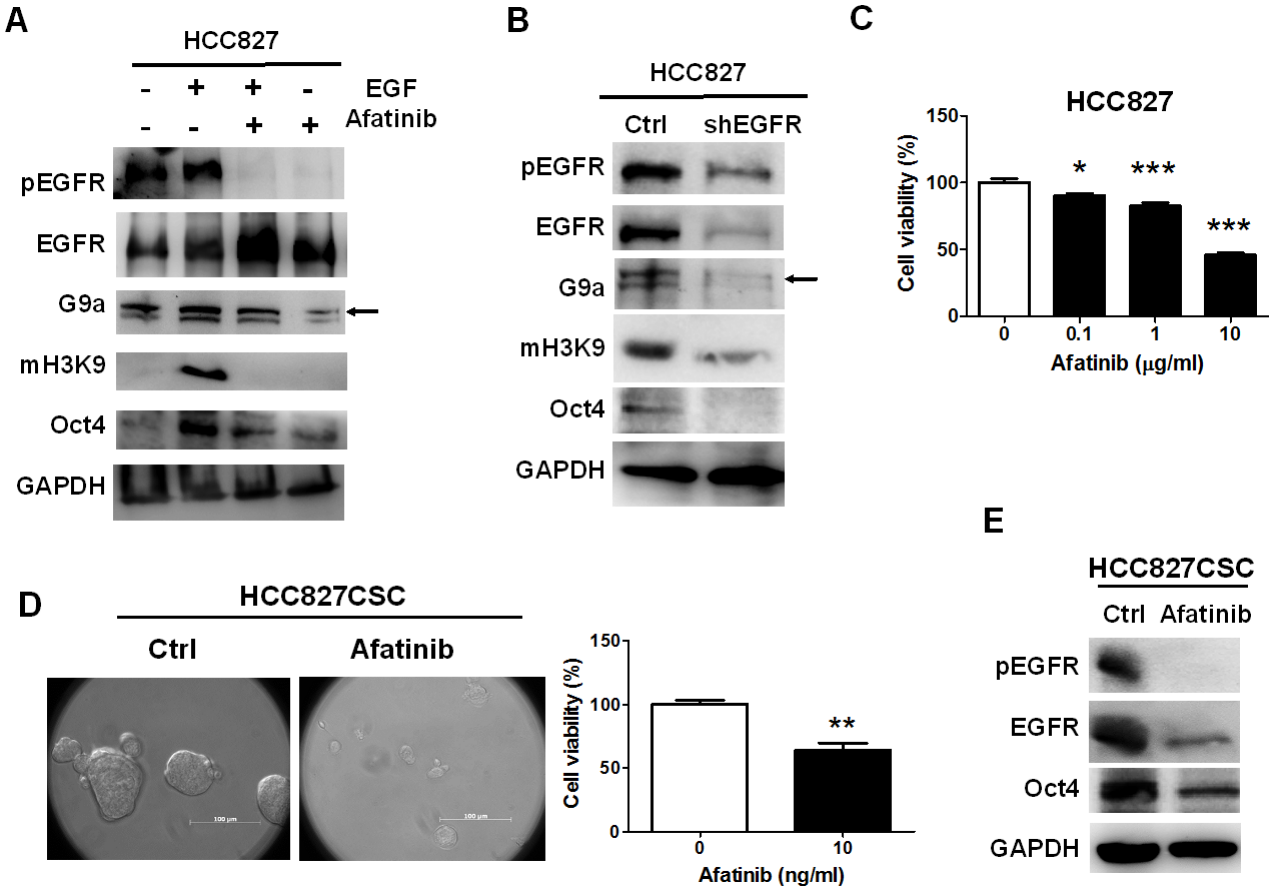
EGFR induced the formation of HCC827-derived tumorspheres and *G9a* expression. To validate that EGFR directly affects the expression of *G9a* and *Oct4* involved in tumorsphere formation, we inhibited EGFR by using afatinib and genetic knockdown to demonstrate the EGFR-G9a regulatory relationship. (A) Afatinib treatment with or without 20 ng/mL of EGF blocked *G9a* expression and H3K9 methylation, accompanied by the reduction in *Oct4* expression, indicating that EGFR positively regulated *G9a* in HCC827 cells. (B) EGFR knockdown with lower EGFR autophosphorylation also reduced *G9a* expression, H3K9 methylation, and *Oct4* expression. (C) To validate whether the blockade of EGFR phosphorylation can reduce tumorsphere formation, afatinib was used. First, afatinib was treated with parental HCC827 cells at a dose of more than 1 μg/mL, which led to a reduction in the viability of HCC827 cells. (D) However, afatinib (10 ng/mL) clearly inhibited the formation of HCC827-derived tumorspheres by approximately 50% (n = 3), revealing that afatinib preferably inhibited cancer stemness. (E) Moreover, afatinib reduced EGFR autophosphorylation and expression and *Oct4* expression in the HCC827-derived tumorspheres, implying that EGFR phosphorylation self-regulated EGFR expression in HCC827 cells. Scale bar: 100 μm. *p < 0.05. **p < 0.01,***p < 0.001.

EGFR phosphorylation was higher in HCC827-derived tumorspheres and EGF induced the expression of G9a and Oct4, implying that EGFR participated in tumorsphere formation. Therefore, to evaluate whether EGFR plays a pivotal role in tumorsphere formation, we blocked EGFR phosphorylation using afatinib. Afatinib was treated with or without EGF in HCC827 cells. We observed that 20 ng/mL of EGF induced the expression of *G9a* and *Oct4* in HCC827 cells (Fig 3A), which was suppressed by afatinib (Fig 3A). Compared with YM155, afatinib inhibited the phosphorylation of EGFR in EGF co-treated cells, resulting in a marked reduction of the methylation on H3K9 (Figs 2I and 3A). To specifically ensure that the expression and activity of *G9a* are regulated through EGFR phosphorylation, EGFR was targeted with a knocked down shRNA, which markedly reduced the expression of EGFR, *Oct4*, *G9a*, and *mH3K9* (Fig 3B); this suggests that EGFR regulates the expression and activity of G9a and maintains the stemness property in lung HCC827 cells.

Next, afatinib significantly inhibited the viability of HCC827 cells in a dose-dependent manner (Fig 3C), and it also reduced tumorsphere formation at a dose of 10 ng/mL (Fig 3D), leading to an approximately 50% reduction in cell viability, similar to the effects of afatinib on parental HCC827 cells (Fig 3C and 3D). To ensure that the tumorsphere reduction was engendered from the inhibition of the stemness property, afatinib-treated tumorspheres were collected and analyzed through Western blotting for measuring *Oct4* expression. We observed that afatinib blocked EGFR autophosphorylation and also reduced *Oct4* expression in HCC827 CSCs (Fig 3E).

### G9a determined EGFR-mediated stemness in lung cancer cells

**Fig 4:**
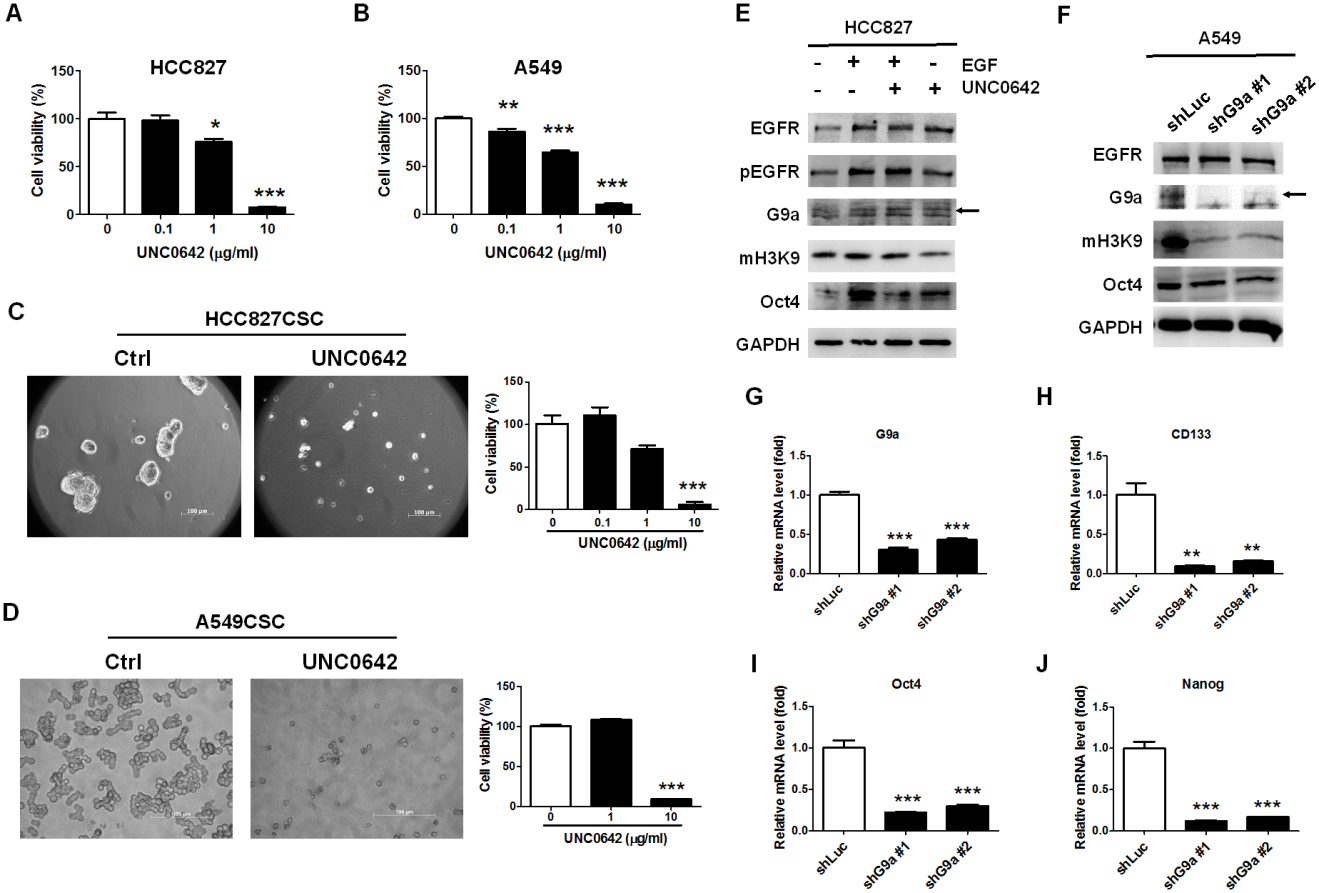
G9a–GLP inhibitor, UNC0642, and G9a knockdown inhibited G9a-mediated H3K9 methylation and reduced stemness in lung cancer cells. To determine that *G9a* is a regulator of stemness in lung cancer, the G9a–GLP inhibitor and genetic knockdown of G9a were applied to inhibit the formation of tumorspheres and expression of stemness markers, respectively. (A) We found that UNC0642 significantly reduced HCC827 cells when used at a dose of less than 1 μg/mL and (B) A549 cells when used at a dose of less than 0.1 μg/mL. (C) UNC0642 also significantly reduced the formation of HCC827 CSCs when used at a dose of 10 μg/mL and (D) A549 CSCs. The reduction in cell viability was similar to that observed for parental cells. (E) Therefore, to validate that the reduction in tumorsphere formation was derived from the inhibition of the cancer stemness property, *Oct4* was detected in UNC0642-treated HCC827 cells with or without 20 ng/mL of EGF. We found that UNC0642 reduced EGF-mediated mH3K9 and *Oct4* expression in HCC827 cells. (F) To validate the significant role of G9a in regulating the stemness property, we knocked down G9a by using shRNA technique and investigated the methylation of H3K9 and expression of *Oct4* in EGFR wild-type A549 cells. We found a marked reduction in H3K9 methylation and a slight reduction in *Oct4* expression in the *G9a* knockdown A549 cells. (G) The inhibition of *G9a* expression led to a reduction in the mRNA levels of (H) *CD133*, (J) *Oct4*, and (K) *Nanog*, indicating that *G9a* played a significant role in regulating the stemness property of lung cancer cells. Scale bar: 100 μm. *p < 0.05. **p < 0.01. ***p < 0.001.

To understand the role of G9a in tumorsphere formation, we treated HCC827 and A549 cells with UNC0642, an inhibitor antagonizing *G9a* and *GLP* [29]. We observed that UNC0642 markedly reduced the viability of the cells in a dose-dependent manner (Fig 4A and 4B). Moreover, UNC0642 reduced tumorsphere formation in HCC827- and A549 CSCs (Fig 4C and 4D), with a significant reduction in viability at a dose of 10 μg/mL; the results indicate that G9a plays an important role as the downstream protein in the cancer stemness activity. Furthermore, to validate that G9a regulates the stemness property in lung cancer and understand whether it downregulates EGFR signaling in lung cancer cells, UNC0642 was co-treated with or without EGF in HCC827 cells. We found that UNC0642 reduced G9a activity, as indicated by the expression of mH3K9, and inhibited EGF-induced *Oct4* expression (Fig 4E). In addition, we knocked down G9a by using a specific shRNA technique in A549 cells and found that G9a reduction resulted in the downregulation of mH3K9 and *Oct4* (Fig 4F). *G9a* knockdown, as confirmed by measuring the mRNA levels (Fig 4G), resulted in the reduction in the mRNA levels of *CD133* (Fig 4H), *Oct4* (Fig 4I), and *Nanog* (Fig 4J), suggesting that G9a determined the cancer stemness property in A549 cells. These results reveal that YM155 reduced tumorsphere formation by inhibiting the EGFR-mediated activation of *G9a* in the cancer initiation program of HCC827 and A549 CSCs.

## Discussion

CSCs are the main reason for tumor recurrence. In this study, we demonstrated that EGFR triggered the formation of tumorspheres derived from HCC827 and A549 cells, which expressed *CD133*, *Oct4*, and *Nanog* used as the cancer stemness models. We observed that the potential EGFR-G9a pathway played an essential role in the formation of lung CSCs. This study also revealed that YM155 simultaneously inhibited the autophosphorylation of EGFR and G9a-mediated stemness property in HCC827 and A549 cells, thus rendering it an efficient anti-stemness inhibitor.

According to our review of the relevant literature, EGFR overexpression exceeds 90% in lung cancer. Therefore, targeting EGFR is considered an efficient therapeutic strategy against lung cancer, particularly for inhibiting EGFR phosphorylation by using gefitinib and afatinib. However, the mutations of EGFR-downstream proteins, such as KRAS in A549, result in drug resistance, protecting cancer cells from therapeutic treatments [18]. Moreover, the therapeutic inhibition of EGFR induces gene arrangement and acquired drug resistance against TKIs [30,31]. These drug resistance strategies include the induction of other oncogenic drivers, such as the induction of T790M mutations in the EGFR gene [32], MET [33], or HER2 amplifications [34]. Drug resistance is highly associated with CSC development [35,36]. The major downstream regulators determining cancer stemness are derived from either EGFR signaling or another drug-resistant pathway; therefore, they have been considered as therapeutic targets to overcome the acquired resistance from cancer stemness.

G9a, an SET domain-containing HMT, interacts with GLP to form heterodimers through the SET domain [37]. G9a-GLP methylates on H3K9 and H3K27 [38]. H3K9 methylation is considered to condense the genome chromatin and inhibit DNA transcription, thus indicating that G9a is a transcriptional corepressor. In addition to methylation on histones, G9a-GLP methylates nonhistone proteins [39]. G9a was revealed to activate p53 through a methylation-independent mechanism [40]. G9a was also reported to methylate p53 at Lys373, which is associated with p53 activity [39]. Additionally, a study revealed that G9a positively regulated gene expression by recruiting GRIP1, CARM1, and p300 [41]. Therefore, we speculate that G9a acts as a coactivator under some cellular conditions instead of a gene repressor. However, the detailed mechanism of G9a in determining cancer stemness remains unclear. In this study, we found that *G9a* inhibition and knockdown by UNC0642 or an shRNA technique, respectively, reduced *Oct4* expression, implying that *G9a* induced *Oct4* expression as a co-activator. The results are not in concordance with the literature that *G9a*-induced methylation on H3K9 results in *Oct4* repression [42]. Since *Oct4* is an important transcriptional factor for regulating the pluripotency of embryonic stem cells [43], and *G9a* is essential for early embryogenesis [21]. Therefore, we believe that *G9a* overexpression in CSCs preferably positively regulates *Oct4* for the developmental transition of cancer stemness. However, the detailed mechanism of *G9a* in regulating cancer stemness warrants further investigation.

In their cohort study, Rada et al indicated *G9a* to be associated with a higher survival rate among patients with early lung cancer [40]. However, *G9a* was reported to be elevated and associated with tumor invasion, leading to a poor survival rate among patients with lung cancer [44]. This result is in concordance with our findings based on the TCGA database in this study (Fig 2I). Notably, the status of CSCs is different from that of well-grown cancer cells; the cells are tolerant to treatments with slower growth capacity. Therefore, the artificial low attachment in serum-free medium mimics a stressful environment that enforces the cells to develop temporary stemness properties. *G9a* was demonstrated to be upregulated in many types of cancers [39] and is probably involved in the transition of stemness, thus resulting in a poor survival rate in clinical practice. A previous study revealed that *G9a* knockout could reduce embryonic cell growth in G9a^−/−^ mutant mice [21], indicating that *G9a* is essential for early embryogenesis. In this study, we validated that cancer stemness, as measured using *Oct4* expression, was regulated by *G9a* in the temporary stemness status derived from EGFR-positive lung cancer cells, which was the EGFR-downstream protein serving as a therapeutic target.

We observed that 10 ng/mL of YM155 more significantly inhibited the formation of HCC827-derived CSCs than did afatinib and UNC0642. YM155, an imidazolium-based survivin-suppressing compound [10], binds and inhibits ILF3 [9]; this thus suggests that ILF3 may contribute to the EGFR-mediated stemness property in HCC827 cells. The detailed mechanism and function of ILF3 warrant investigation in the future. Studies have reported that YM155 can inhibit tumorsphere formation [6,45]. In addition, YM155 has many functions and inhibits at least EGFR and survivin, thus serving as an anti-tumor agent [5,6]. In this study, we observed that YM155 inhibited EGFR autophosphorylation and *G9a* expression, rendering it a potent agent against lung cancer stemness.

In conclusion, EGFR phosphorylation was high in HCC827- and A549-derived tumorspheres, and this phosphorylated EGFR positively regulated *G9a* expression. *G9a* activity was associated with *Oct4* expression, indicating that EGFR mediated the upregulation of *Oct4* through *G9a*. Moreover, we observed that inhibitors, namely YM155, afatinib, and UNC0642, efficiently reduced tumorsphere formation. YM155 had a stronger inhibitory capacity against cancer stemness by simultaneously inhibiting EGFR autophosphorylation and *G9a* expression. Our data strongly suggest that YM155 as an inhibitor of lung cancer stemness is a potent agent against lung cancer.
